# Can TSH level and premenstrual spotting constitute a non-invasive marker for the diagnosis of endometriosis?

**DOI:** 10.1186/s12905-021-01474-3

**Published:** 2021-09-20

**Authors:** Lena Birke, Dunja M. Baston-Büst, Jan-Steffen Kruessel, Tanja N. Fehm, Alexandra P. Bielfeld

**Affiliations:** 1 Department of OB/GYN and REI (UniKiD), Medical Center, Medical Faculty, University of Düsseldorf, Moorenstrasse 5, 40225 Düsseldorf, Germany; 2 Department of OB/GYN, Medical Center, Medical Faculty, University of Düsseldorf, Moorenstrasse 5, 40225 Düsseldorf, Germany

**Keywords:** Dysmenorrhea, Dyspareunia, Endometriosis, Infertility, Marker, Thyroid dysfunction

## Abstract

**Background:**

To date, there is no reliable non-invasive marker for the early detection and diagnosis of endometriosis available possibly resulting in a delayed diagnosis and consequently an unnecessary long ordeal for the individual woman. Therefore, the primary objective of the current study was to evaluate whether the combination of a thyroid-stimulating hormone (TSH) level > 2.5 µlU/ml and premenstrual spotting could serve as non-invasive markers of endometriosis. A secondary objective was to determine whether typical symptoms of endometriosis like dysmenorrhea and/or dyspareunia could increase the diagnostic reliability.

**Methods:**

We conducted a retrospective, case–control study with 167 female patients at the Department of OB/GYN and REI (UniKiD) of the medical center of the University of Düsseldorf, between January 2015 and December 2016. 107 women with surgically confirmed endometriosis were compared to 60 without endometriosis (controls). To evaluate the diagnostic accuracy, we considered sensitivity, specificity and predictive values. In order to assess the association between the non-invasive markers and endometriosis an odds ratio (OR) with a 95% confidence interval was calculated.

**Results:**

In our cohort, diagnosis of endometriosis with non-invasive markers according to their sensitivity yielded the following ranking: increased TSH level, premenstrual spotting, combination of both previous parameters, addition of dysmenorrhea, addition of dyspareunia and combination of all parameters.

**Conclusion:**

The existence of endometriosis should be taken into consideration when a patient suffers from thyroid dysfunction and premenstrual spotting. Apart from an increased TSH level, the presence of premenstrual spotting underlines the possible diagnosis of endometriosis with non-invasive markers and therefore, the patient´s history needs to be taken into account carefully.

*Trial registration* The retrospective study was approved by the Ethics Committee of the medical faculty of the Heinrich-Heine University, Düsseldorf, Germany, Registration number Düsseldorf: 5371R (approved: April 04th, 2016). Since the design of the study was retrospective no written informed consent was necessary.

## Background

Endometriosis is a common benign and chronic inflammatory disease in women that is characterized by the presence of endometrial tissue outside the uterine cavity. About 10% of all reproductive-aged women are affected [1]. Endometriosis is frequently associated with chronic pelvic pain, dysmenorrhea, dyspareunia and irregular uterine bleeding [2, 3]. The prevalence of endometriosis in women with infertility has been estimated to be up to 30–56% [4].

The diagnostic gold standard until today is the combination of laparoscopy and the histological verification of endometrial glands and/or stroma in the biopsies [5]. Potential complications of the surgical procedure are vascular injuries, anesthetic complications, damage to bowel, bladder and uterus, thromboembolism and wound infections [6]. Furthermore, the diagnosis of endometriosis tends to be strikingly delayed. Time-lags from 6.7 to 10.4 years between the first onset of clinical symptoms until surgical confirmation have been observed [7–9]. In addition to the impaired quality of life, there are increased health care costs for physician visits, surgery and hospitalization and loss of work time due to illness [7, 10]. In particular, reported symptoms such as abdominal pelvic pain, dysmenorrhea, menorrhagia, dyspareunia, postcoital bleeding and unwanted childlessness are associated with endometriosis. Especially, when several symptoms are present at the same time [11]. Consequently, the development of non-invasive markers has become a major focus of interest in the diagnosis of endometriosis [12]. The very recent hypothesis, that an abnormal intestinal permeability might play a role in the pathogenesis of endometriosis is therefore very interesting not only to understand the pathogenesis better but also as a possible non-invasive marker [13]. In general, exploring new techniques like metabolomics in the diagnosis of endometriosis seems to be a promising new approach by identifying metabolic alterations in endometriosis patients [14]. The possibility to discover those changes by means of a simple serum test makes it a promising approach. However, it is noteworthy, that a clinical examination and vaginal ultrasound are of course still very important tools in the comprehensive clinical examination.

So far several groups reported an association between endometriosis and thyroid dysfunction [15–19]. However, thyroid-stimulating hormone (TSH) levels were considered seldom in the literature. Nevertheless, it was already described that TSH acts as a proliferative hormone on endometria of patients resulting in being an interesting target in the search of a non-invasive marker. So far, autoimmune thyroiditis has been investigated more closely and the prevalence of positive antibodies could be associated with endometriosis [16, 19].

Heitmann et al. reported a link between premenstrual spotting and histologically confirmed endometriosis and subsequently suggested that premenstrual spotting can be a marker in the diagnosis of endometriosis [20].

Up until now a combination of both markers—TSH and premenstrual spotting—was not considered as a diagnostic tool. Thus, the purpose of this study was to evaluate, whether a combination of the aforementioned two markers as well as dysmenorhea and dyspareunia as common markers for endometriosis can increase the accuracy of a non-surgical diagnosis.

## Methods

This retrospective case–control study was conducted with 167 patients (107 (64%) cases and 60 (36%) controls) at the Department of OB/GYN and REI (UniKiD) of the Medical Center of the University of Düsseldorf. The study has been approved by the Ethics Committee of the Heinrich-Heine University Düsseldorf (5371R [2016-04-04]). No written consent was necessary according to the Ethics Committee. Women who consulted the center, were included for infertility treatment between January 2015 and December 2016. The age ranged from 22 to 44 years, with a mean age of 35.38 years.

**Fig. 1:**
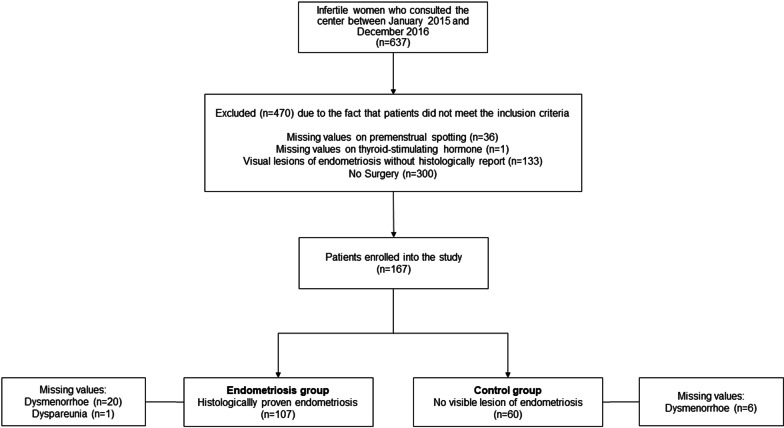
Flowchart of study population selection

From the initially 637 patients included, 470 did not meet all inclusion criteria. Inclusion criteria for the endometriosis group (EG) were a histologically confirmed endometriosis and for the control group (CG) the surgical exclusion of endometriosis (Fig. 1).

Primary parameters of interest were serum TSH levels (blood was taken between 8 and 10 am) and premenstrual spotting. The recommended TSH level before pregnancy is < 2.5 µIU/L [21]. Patients with a basal TSH value > 2.5 µlU/ml started with levothyroxine. Forty four percent (73/167) of the women already took levothyroxine medication at the initial visit. Therefore, we stratified two different groups: TSH level above 2.5 µlU/ml (with/without intake of levothyroxine) and TSH level less than 2.5 µlU/ml with an intake of levothyroxine were considered as patients with thyroid dysfunction. Patients with TSH level less than 2.5 µlU/ml without an intake of levothyroxine were regarded as thyroid-healthy.

Premenstrual spotting was defined as a light bleeding for 1–3 days before the onset of the menstrual flow. Patients were asked specifically for spotting, dysmenorrhea (abdominal pain/cramps during the menstrual flow) and dyspareunia (painful sexual intercourse). No information could be obtained from 20 patients in the EG and 6 patients in the CG concerning dysmenorrhea. Data about dyspareunia was missing in 1 case in the EG (Fig. 1). Additional information was adopted from patients´ records including age, smoking, length of the menstrual cycle, number of pregnancies, life births und number of pregnancy loss. The information was collected from physicians at the initial visit by means of a structured questionnaire. Data concerning the severity of endometriosis according to revised American Society of Reproductive Medicine (rASRM) score are shown in Table 1 [22].

**Table 1:** Baseline characteristics of patients included in the study, endometriosis group (EG) vs. control group (CG)

Characteristics	EG (n = 107)	CG (n = 60)	*P* value^f^
Age^a^	35.34 ± 3.48(107), median = 35	35.47 ± 4.62 (60), median = 36	> 0.05
*rASRM endometriosis score (n)*			
1	27		
2	40		
3	28		
4	12		
*Smoker*^b^			
Yes	16% (17)	17% (10)	> 0.05
Menstrual cycle length^a^	28.27 ± 2.23 (97) median = 28	28.18 ± 2.10 (48) median = 28	> 0.05
Pregnancies^c^	30% (32)	42% (25)	> 0.05
Life births^d^	9% (10)	22% (13)	< 0.05
Abortions^e^	22% (24)	33% (20)	> 0.05

^a^Data are presented as mean values ± standard deviations (absolute incidence), median

^b^Data are presented as percentage (absolute incidence)

^c^Number of patients who were pregnant before starting infertility treatment

^d^Number of patients who gave birth before starting infertility treatment

^e^Number of patients who had miscarriage(s) before starting infertility treatment

^f^A *P* value < 0.05 was considered significant

The primary hypothesis (abbreviated as “H” and numbered) of this study was: (H1) Can the presence of endometriosis be derived from a TSH level > 2.5 µlU/ml in combination with premenstrual spotting?

Secondarily, the following hypotheses were tested:

Is there an association between endometriosis and:

- (H2) a TSH level > 2.5 µlU/ml, premenstrual spotting as well as dysmenorrhea,
- (H3) a TSH level > 2.5 µlU/ml, premenstrual spotting and dyspareunia,
- (H4) a TSH level > 2.5 µlU/ml, premenstrual spotting, dysmenorrhea and dyspareunia,
- (H5) premenstrual spotting alone,
- (H6) thyroid dysfunction alone.

The descriptive statistical analysis for sensitivity, specificity, positive and negative predictive value and accuracy were calculated for the TSH levels, premenstrual spotting and the symptoms of dysmenorrhea and dyspareunia. We also calculated an OR with 95% confidence intervals (Microsoft Excel 2019). Baseline characteristics between the different groups were compared using the two-tailed Student’s t test and Levene’s test (comparison of means) for analysis of continuous variables as well as Pearsonʼs Chi-Quadrat-test for categorical variables (IBM SPSS Statistics Version 27). A *P* value < 0.05 was considered statistically significant.

## Results

The baseline characteristics of the 167 study participants are shown in Table 1 without any statistically significant differences between the groups with exception of the number of women who gave birth before starting infertility treatment. The primary aspect of interest was premenstrual spotting and serum TSH level.

51% patients in the EG and 22% in the CG reported premenstrual spotting. TSH level was 3.21 ± 1.89 µlU/ml in the EG vs 2.52 ± 1.31 µlU/ml in the CG for women with an intake of levothyroxine and 1.78 ± 0.71 µlU/ml in the EG vs 1.68 ± 0.65 µlU/ml in the CG for women without an intake of levothyroxine.

In the EG, 69% patients reported dysmenorrhea and 32% dyspareunia. Of the 60 women in the CG, 37% reported dysmenorrhea and 7% dyspareunia (Table 2).

**Table 2:** Clinical aspects in patients with (EG) vs. without (CG) endometriosis

Parameters	EG (n = 107)	CG (n = 60)
Premenstrual spotting^b^	51% (55)	22% (13)
Dysmenorrhea	69% (60)	37% (20)
Missing value	19% (20)	0.1% (6)
Dyspareunia	32% (34)	7% (4)
Missing value	0.9% (1)	
TSH level^a^	3.21 ± 1.89 (47)^c^	2.52 ± 1.31 (26)^c^
	1.78 ± 0.71 (60)^d^	1.68 ± 0.65 (34)^d^

TSH: Thyroid-stimulating hormone

^a^Data are presented as mean values ± standard deviations (absolute incidence), median

^b^Data are presented as percentage (absolute incidence)

^c^Women with intake of levothyroxine

^d^Women without intake of levothyroxine

The tested hypotheses of thyroid dysfunction and premenstrual spotting (H1), thyroid dysfunction, premenstrual spotting and dysmenorrhea (H2) and premenstrual spotting alone (H5) are highly significant associated with the presence of endometriosis (Table 3). The largest effect in predicting endometriosis was seen in the presence of premenstrual spotting (H5). No significant correlation was found between thyroid dysfunction and endometriosis in the EG alone, the combination of thyroid dysfunction, premenstrual spotting as well as dyspareunia (H3) and the combination of all 4 markers (H4) (Table 3).

**Table 3:** Association between the non-invasive markers and endometriosis

Tested hypothesis	Odds ratio	95% CI	*P* value^a^
H1 (n = 167)	2.7546	1.22–6.23	0.0149
H2 (n = 141)	3.3169	1.17–9.38	0.0238
H3 (n = 166)	3.1319	0.87–11.3	NS
H4 (n = 140)	2.2368	0.59–8.53	NS
H5 (n = 167)	3.8240	1.86–7.87	0.0003
H6 (n = 167)	1.0980	0.58–2.07	NS

Data are given as mean and 95% confidence interval

CI: Confidence interval, NS: not significant

^a^A *P* value < 0.05 was considered significant

Regarding the correlation between endometriosis and premenstrual spotting and endometriosis and the TSH level, premenstrual spotting yielded a sensitivity of 51.40%. TSH level yielded a sensitivity of 52.34% (Table 4).

**Table 4:** Performance of premenstrual spotting, TSH level > 2.5 µlU/ml, dysmenorrhea and/or dyspareunia as marker for non-invasive diagnostic of endometriosis

Tested hypothesis	Sensitivity (%)	Specificity (%)	Positive predictive value (%)	Negative predictive value (%)	Accuracy (%)
H1 (n = 167)^a^	32.71	85	79.55	41.46	51.50
H2 (n = 141)	25.29	90.74	81.48	42.98	50.35
H3 (n = 166)	14.15	95	83.33	38.51	43.37
H4 (n = 140)	11.63	94.44	76.92	40.16	43.57
H5 (n = 167)	51.40	78.33	80.88	47.47	61.08
H6 (n = 167)	52.34	50	62.12	37.04	51.50

^a^Data are presented as percentage

Both markers (H1) could correctly classify 51.50% of the women, yielding to a sensitivity of 32.71%.

Combining the markers dysmenorrhea and dyspareunia, the sensitivity for the different groups (H2–H4) differed and were overall low with 25.29% for H2, 14.15% for H3 and 11.63% for H4 (Table 4).

## Discussion

Applying both markers, increased TSH and premenstrual spotting resulted in a correct diagnosis of endometriosis in 52% of the women in our cohort. Adding symptoms as dysmenorrhea and dyspareunia did not increase the diagnostic accuracy substantially. Endometriosis was correctly classified in 50% of the women with thyroid dysfunction, premenstrual spotting and dysmenorrhea. Considering premenstrual spotting alone, endometriosis was correctly classified in 61% of the women.

To our knowledge, this is the first study to assess a combination of the non-invasive markers TSH level and premenstrual spotting for the precise non-invasive prediction of endometriosis prediction. The advantages of these markers are that they are simple to obtain and easily available. Therefore, premenstrual spotting and the TSH level should be routinely incorporated in the patient’s history.

Previous studies reported that premenstrual spotting is associated with endometriosis [20, 23–25]. The exact cause of premenstrual spotting in women with endometriosis is unknown though. A possible explanation is that they are more often affected by luteal phase deficiency [26]. In our group, women with endometriosis had a higher prevalence of premenstrual spotting (51%) compared to controls (22%). The results of the present study differ from those reported by Heitmann et al. [19] who detected premenstrual spotting in 89% (34/38) of women with endometriosis vs 11% (4/42) in controls and had a sensitivity of 76% and specificity of 90% which is higher than in our investigation. However, this discrepancy could be explained by the smaller number of patients who were enrolled. Moreover, Heitmann et al. analyzed 80 consecutive patients consulting an infertility clinic [20].

In contrast to the latter study, but comparable to our data, a retrospective observation showed 32% (106/332) of women with endometriosis reporting premenstrual spotting.By comparison, only 12% (42/341) of patients in the CG were affected. With 673 women, the number of patients was larger than our group size [23].

Already in the 1980s, an association between premenstrual spotting and endometriosis was found in a study with 55 infertile women. 35% (8/23) had premenstrual spotting while in the comparison group only 6% (2/32) of patients reported premenstrual spotting [25]. A prospective questionnaire-based study with 1200 women found no statistically significant difference between the groups in relation to premenstrual spotting [24]. A limitation of the aforementioned study though is the fact, that the patients answered the questionnaire without medical guidance. Therefore, it is possible that some individuals may have misinterpreted questions or misunderstood the name of specific symptoms or diseases. In our study, symptoms were asked by trained physicians which surely is an advantage.

Concerning TSH level alone and the presence of endometriosis, no significant difference could be found in our study (OR = 1.1, 95% CI 0.58–2.07).

Similar results were reported by Petta et al. [27]. They did not find a significant correlation between hypothyroidism, hyperthyroidism and autoimmune thyroid disease in patients with endometriosis.

Until today, the Sampson theory of the endometriosis pathogenesis is a widely accepted hypothesis, based on observation that endometriotic implants in the pelvis result from retrograde menstruation of endometrial tissue through the fallopian tubes. This theory though is certainly not the only explanation nor does it explain all sites of endometriosis deposits suggesting a contribution of other factors. A field that gained compelling attention recently is the area of stem cells: here in particular the role of stem cells and their involvement in the pathogenesis of endometriosis. As it is currently published, the endometrium contains stem/progenitor cells responsible for the regular regeneration which also play a role in the onset of endometriosis. Those stem cells have the capacity to explain endometriotic lesions in the peritoneal cavity and areas reachable via vascular or lymphatic spread but cannot explain the onset of endometriotic lesions in secluded areas. Here, multipotent stem cells originating from bone marrow (BMDSCs) represent a source of stem cells that can explain remote endometriotic lesions e.g. in the brain and prostate. It is noteworthy, that endometriosis can interfere with the normal recruitment of BMDSCs to the uterus and therewith inducing a dysfunctional endometrium possibly explaining the relatively high number of patients with infertility/subfertility [28, 29].

Genetic factors are believed to play another central role in the pathogenesis of endometriosis. Very recently, new concepts regarding a genetic predisposition emerged. Several single nucleotide polymorphism (SNPs) have been associated with the disease, for example in the region of the wingless-type mammalian mouse tumor integration site family member 4 (WNT4), vezatin (VEZT) and follicle stimulating hormone beta polypeptide (FSHB) [30, 31]. Those genes are known to be involved in molecular mechanisms associated with proliferation and development of endometriotic lesions. In this regard it is of interest, that discrepancies regarding genetic associations due to the individuals’ ethnic background exist, as shown in a study of a Mediterranean population displaying no association of the aforementioned SNPs with the risk to develop endometriosis [32]. Furthermore, current research suggests a link between a disturbed microbiome and the pathogenesis of endometriosis. Particularly gram-negative phyla like Proteobacteria, Bacteroides and Negativicutes of various microbiome sites were increased [33].

Not only bacteria but also cells of the immune system seem to be a player in the onset of endometriosis. Recent findings showed that invariant Natural Killer T-cells (iNKT), a specialized subset of T cells, combining innate and adaptive immunologic characteristics, are capable of secreting Th1 and Th2 cytokines which makes them a candidate for the regulation of endometriosis occurrence [34].

Another new approach is the detection of small bowl permeability by investigation of lipopolysaccharides plasma values and urinary excretion of mannitol and lactulose as non-invasive markers. Significantly higher lipopolysaccharides plasma levels were found in the endometriosis group, whereas the results of mannitol and lactulose were not significant [13]. The application of metabolomics for biomarker discovery is another promising method. Using 1H-nuclear magnetic resonance NMR spectroscopy the group of Murgia and co-workers showed a significant increase in β-hydroxybutyric acid and glutamine, whereas tryptophan was decreased in serum of endometriosis patients [14].

The interest in non-invasive biomarkers is evident in various fields of medicine and findings in one area might be translated to others. Therefore, Fluorescence measurement of erythrocyte zinc protoporphyrin in the lip is a novel interesting non-invasive method for the detection of iron deficiency e.g. The study group by Hennig et al. has succeeded in detecting zinc protoporphyrin without blood sampling in adults and infants for the first time [35, 36]. In the future, it is desirable to find a similar specific marker for endometriosis.

For the diagnosis of non-alcoholic fatty liver disease (NAFLD) or non-alcoholic steatohepatitis (NASH), a liver biopsy was necessary up until now. In a systematic review, microRNAs (miRNAs) in serum and plasma were found “to segregate NAFLD from NASH” [37]. MiRNA-122 showed a diagnostic odds ratio of 9.1 and MiRNA-34a of 6.25 in the diagnosis of NAFLD vs. healthy controls. In our study, comparison of EG with healthy women showed an OR of 2.75 suggesting a greater susceptibility. Interestingly, circulating miRNAs may also be promising candidates for the non-invasive diagnosis of endometriosis as several miRNAs have been identified to be dysregulated [38]. Another recent study suggested that the use of a panel of six miRNAs allows clinicians to differentiate between endometriosis and other gynecologic pathologies [39].

The pain symptoms most commonly attributed to endometriosis are dysmenorrhea, dyspareunia, and chronic pelvic pain. Nevertheless, data concerning the prevalence of dysmenorrhea vary from 68 to 82.5% [23, 24, 40–43], which coincides with our result (69%). Ballard et al. reported the prevalence of dysmenorrhea with only 24.6% in a large cohort study [11]. A possible explanation for these different results is the individual sense of pain. Thus, age, gender, race, cultural background and mental health might influence people in the way how they perceive pain [44].

Additionally, endometriosis may also develop asymptomatically and is sometimes only diagnosed with approaches of infertility or during a laparoscopy for infertility reasons [1, 45, 46]. The symptom dyspareunia has been a common finding in previous studies ranging from 9 to 72% [11, 23, 24, 40–43]. In our study, 32% of women confirmed dyspareunia. As stated above, the different perception of pain may be also of influence here.

Nonetheless, both symptoms may serve as important anamnestic indicators of endometriosis.

In patients with a clinical suspicion of endometriosis, laparoscopy with histological verification is performed to confirm the diagnosis. However, a negative histology does not rule out an endometriosis. On the contrary, endometriosis lesions can also be found in apparently normal peritoneum [47, 48]. With the help of an easily feasible non-invasive method, the diagnosis could be confirmed earlier and prolonged suffering might be avoided. Furthermore, there would be no surgical risks for women without endometriosis who would otherwise need a laparoscopy because of chronic pelvic pain [49]. There is a debate about whether a late diagnosis also implies a progression of the disease which could possibly be prevented by an earlier diagnosis [50, 51]. Furthermore, it needs to be discussed that a late or even too late diagnosis of endometriosis, particularly of ovarian associated endometriomas, might lead to the development of ovarian cancer. A current review highlights, that the risk of developing ovarian cancer on the basis of ovarian endometriosis is stupendous although it is still not clear how much higher the absolute risk is. In this regard it is important to mention, that the majority of endometriosis-related ovarian carcinomas develop in the presence of atypical ovarian endometriosis [52].

Up to date, non-invasive diagnostic methods cannot replace the laparoscopy detection of endometriosis, but the establishment of a non-invasive marker holds a great challenge.

With this work, we would like to point out that the presence of the non-invasive markers premenstrual spotting, thyroid dysfunction and/or dysmenorrhea may indicate endometriosis. It is advisable to sensitize and thoroughly inform the patient about the disease, especially if the patient desire to have children. However, mentioning the presumption of a possible endometriosis might cause anxiety and fear. On the other hand, it might be a relief for many women to be informed about the causes of their symptoms, especially, that there is no serious underlying illness like cancer [53].

The results of our study indicate that the likelihood of suffering from endometriosis is increased if there are several symptoms present at the same time. Therefore, incorporating asking for non-invasive markers like elevated TSH and spotting in the first councelling seems to support an early diagnosis of endometriosis. Future studies might investigate similar or further markers including miRNAs e.g. that might predict endometriosis with non-invasive markers with a good accuracy.

Our study has some limitations: on the one hand is to the retrospective study design and on the other hand missing data for dysmenorrhea 16% (26/167) and dyspareunia 0.6% (1/167). This needs to be considered when interpreting results.

## Conclusions

We found a significant association between endometriosis, premenstrual spotting and the TSH level. The diagnosis of endometriosis should be carefully considered, especially in the presence of premenstrual spotting. Furthermore, in case of infertility, an early consultation of a center for endometriosis and reproductive medicine is advisable.

## Data Availability

The datasets generated and/or analyzed during the current study are not publicly available due to identifying information, but are available from the corresponding author upon reasonable request.

## Abbreviations

TSH: Thyroid-stimulating hormone
EG: Endometriosis group
CG: Control group
rASRM: Revised American society for reproductive medicine
H: Hypothesis
BMDSC: Bone marrow-derived stem cell
SNP: Single nucleotide polymorphism
NAFLD: Non-alcoholic fatty liver disease
NASH: Non-alcoholic steatohepatitis
miRNA: Micro RNA
